# Reported best practices for effective risk management of medical devices: a scoping review

**DOI:** 10.3389/fmedt.2026.1863808

**Published:** 2026-06-24

**Authors:** Rosario Luxardo, Arkeliana Tase, Florence Leong, Massimo Micocci, Martina Tognini, Peter Buckle, Melody Ni

**Affiliations:** 1 Department of Surgery and Cancer, Faculty of Medicine, Imperial College London, London, United Kingdom; 2 NIHR HealthTech Research Centre in In Vitro Diagnostics, London, United Kingdom; 3 Health and Technology Theme, School of Convergence Science, Imperial College London, London, United Kingdom; 4 Biomedical Research Centre, Biomedical Engineering Theme, Department of Bioengineering, Imperial College London, London, United Kingdom

**Keywords:** best practices, *in vitro* diagnostics, intended user, medical device, medical device regulation, patient safety, risk management, standards of care

## Abstract

Risk management (RM) is an essential element for developing safe technologies and frequently a requirement by the regulators. The practice of RM spans from pre-to-post market stages, starting from product development and continuing after product launch. Technological advancements and shifting demands in healthcare led to rapid growth of new technologies such as AI, robotics and point of care diagnostics, presenting many unknowns to the RM process. This study aims to review best practices in medical device RM, highlight gaps and ambiguities in knowledge and identify priorities for future research. A thorough literature review was conducted in June 2025 in two parts: (A) assessment of official updated regulatory documents and guidelines for Medical Devices (MDs), and (B) review published literature for reported best practices in RM of MDs. Part A of the search focused on official regulatory agencies, and Part B of the search was conducted on OVID, Embase, Medline, PubMed, Engineering Village, Scopus, and Google Scholar. A 10-year and English as language limit was applied only for part B. Our search found 147 publications (16 were guidelines from regulatory agencies and 131 were from peer reviewed literature). A total of one regulatory document and 11 studies met the inclusion criteria. Step (A) ISO 14971 was the only regulatory document that explicitly described a structured process for RM. Step (B) Although no single best practice was identified, several recurring themes emerged. Significant best practices suggest the use of hybrid or multi-component frameworks (*n* = 5), the integration of qualitative and structured quantitative approaches (*n* = 3), incorporation of patient preferences (*n* = 2), inclusion stakeholder and end-user engagement (*n* = 1), and considerations of emerging system-level challenges, such as cybersecurity, was limited. Based on our findings, we advise targeted recommendations to strengthen current practices. In conclusion, many challenges exist in conducting an efficient RM process for medical devices, driven by variability in technology and specifications, which will increase as these technologies continue to evolve. Greater end-user involvement throughout all stages can help identify unknown hazards and improve medical technology safety.

## 1 Introduction

Medical devices (MDs) and *in vitro* diagnostic devices (IVDs) are key drivers of economic growth worldwide. The global market for MDs is expected to reach nearly $887 billion by 2032 (1). The UK MedTech industry had a turnover of £34 billion during 2021–2022, with research and development playing an essential role in progress of technology (2). MDs (e.g., robotic surgeries) featured heavily in the UK NHS 10-year plan to deliver sustainable and high-quality care.

A MD is any product intended for use in humans to diagnose, monitor, prevent, or treat a disease. An IVD medical device is a MD intended to generate diagnostic information through the examination of human specimens such as blood or tissue (3).

Over the past decade, the global MD market has expanded rapidly, driven by technological innovations such as integrated AI devices, robotics, and point-of-care test IVDs. Demographics shifts, including an ageing population and divergent healthcare demands further pose significant challenges for manufacturers in meeting regulatory demands and managing risks effectively (4–6). For instance, wearable fitness digital health products monitoring smartwatches may be exempted from MDs regulations requiring less stringent risk management (RM) plans (7). However, as technologies increasingly interact, the reliability and safety of interconnected devices directly impact patient safety and outcomes (8, 9).

Effective RM is essential, as failures of MDs can potentially result in injury and even death (10). MDs should not fail frequently and when they do, these failures must be detected, documented and remedial actions taken to mitigate future risks (11). However, the World health Organisation estimates that 50%–80% of failed equipment remains non-functional due to inadequate maintenance, limited competency and focus on correction rather than prevention (11). Our previous studies with clinical teams in hospital settings confirm this trend, with failure rate as high as 58% for high-risk devices (12, 13).

ISO 14971:2019 is the most widely adopted guideline on RM (14). It defines RM as a systematic application of management policies, procedures and practices to the tasks of analysing, evaluating, controlling and mitigating risk (14). ISO 14971:2019 is adopted by major regulatory agencies across the world and is considered a strategic pillar for successful health innovations facilitating regulatory approval and chances of market viability through structured RM process.

Given increasing technological complexity and the absence of a unified preventive framework, RM is now viewed as a strategic enabler for safe and effective device development. RM spans the entire product lifecycle—from design to post-market surveillance, adding to its complexity (15) Early and continuous engagement with RM, even when data are incomplete, helps developers anticipate regulatory evidence requirements, reducing redesigns, delays, and costs. Conversely, neglecting early RM often leads to inefficiencies and costly modifications (16, 17).

Despite ISO 14971, there is a lack of detailed instructions for demonstrating safety and effectiveness (15). Manufacturers must establish acceptable criteria, resulting in varied processes and performance across similar devices.

These potential risks are not unrecognised by users. Lay advisors from our Patient and Public involvement (PPI) group) at Imperial College London identified three top risks associated with MDs. The top risks identified where: real world usability, regulatory and oversight gaps, and psychological and care burden. They raised concerns that registration of MDs is not as stringent as for drugs and the lack of end user involvement in improvement of device design. Device malfunction adds a “mental burden” to the patient and carers. Additionally, ethical concerns regarding use of AI and inequalities in availability of UK NHS resources were also raised.

In view of technological, regulatory and socio-economic challenges, in this paper we aim to systematically identify and map best practices in medical device risk management through a scoping review combining regulatory guidance and published evidence. Given majority of the MD manufacturers are small to medium enterprises, as well as the critical role that effective RM plays in bringing safe and innovative devices to market, we believe practical guidance is an essential step to implement effective RM. By highlighting gaps and ambiguities in existing frameworks, we seek to inform on the pitfalls and underline points for future research.

## 2 Methods

**Figure 1:**
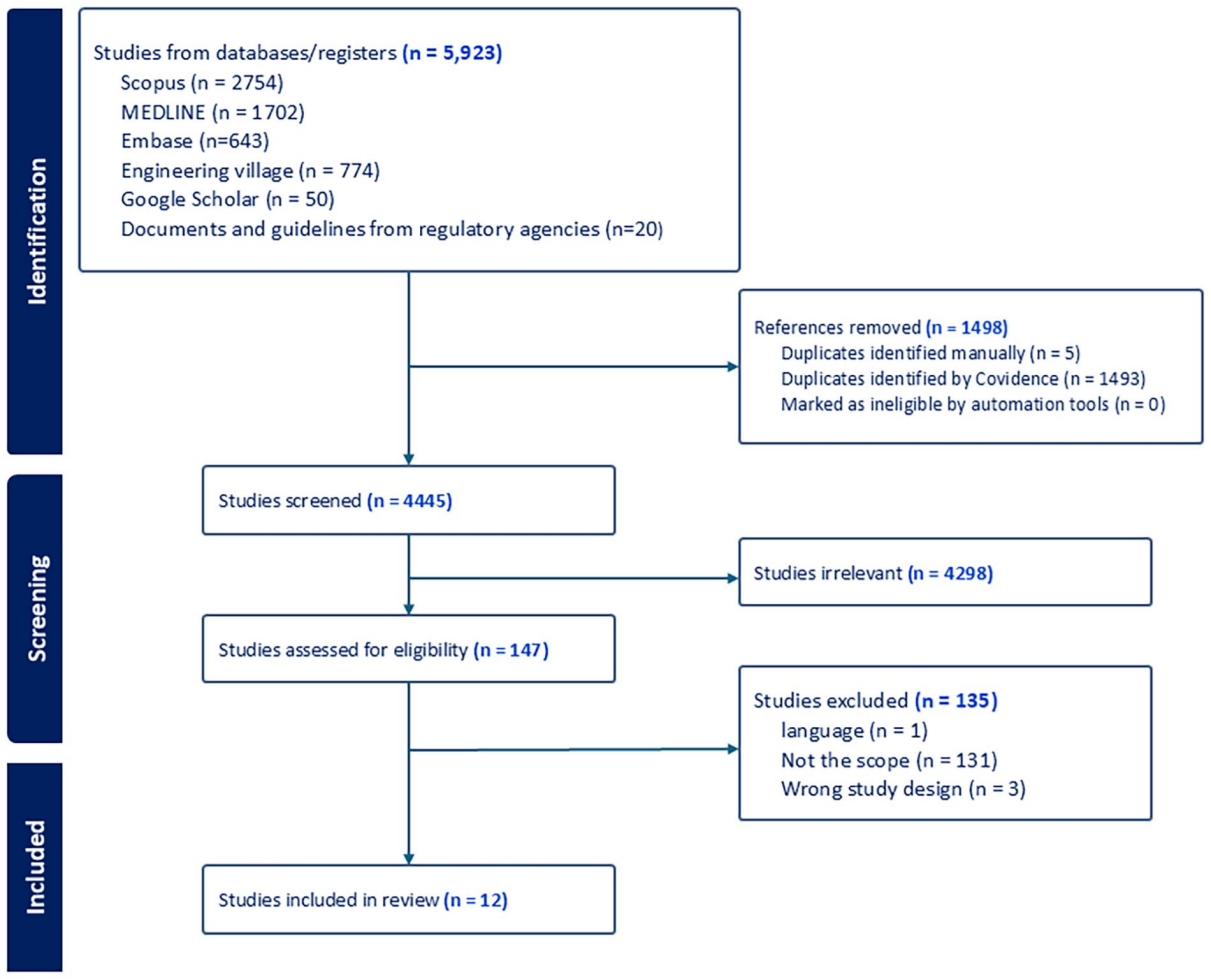
PRISMA flow diagram of study selection process.

A literature search was conducted in June 2025 using OVID Embase and Medline, PubMed, Engineering Village, Scopus and Google Scholar, and regulatory agency websites (more details below in point A) The Key words and MESH terms used included “Risk Reduction Behaviour” OR (“Risk Evaluation and Mitigation”) OR (“Risk management”) OR (“Risk Assessment”) AND (medical device) OR (“Equipment and Supplies”). At the same time, we reviewed guidelines for both MDs and *in vitro* diagnostics. This study was conducted and reported in accordance with the Preferred Reporting Items for Systematic Reviews and Meta-Analyses extension for Scoping Reviews (PRISMA-ScR) guidelines (18). The PRISMA flowchart is shown in Figure 1. The full PRISMA-ScR checklist and search strategies are provided in Supplementary Material.

### 2.1 Search sources

We structured the search in two parts:

- Assessment of official updated regulatory documents and guidelines for MDs from regulatory agencies focused on The Medicines and Healthcare products Regulatory Agency (MHRA), Food and Drug Administration (FDA), International Medical Device Regulators Forum (IMDRF), World Health Organization (WHO), European Medicines Agency (EMA), and International Organization for Standardization (ISO).
- Assess published literature for reported best practices in RM of MDs.

### 2.2 Inclusion/exclusion criteria for the literature review

For part B we included all papers published in English in the last 10 years that provided evidence of RM models in health care. The full inclusion and exclusion criteria for the literature search can be found in Table 1.

**Table 1:** Inclusion and exclusion criteria .

Inclusion criteria	Exclusion criteria
Regulations or guidelines for MDs currently applicable or relevant to UK	Articles not published in English
Expert recommendations or best practices applicable to MDs published literature between January 2014 to May 2025	Articles published before 2014

### 2.3 Search results management

All citations were uploaded to Covidence UK 2025, and duplicates were removed. Title and abstract screening were carried independently by two reviewers (RL and AT) based on inclusion criteria. Full text manuscripts were assessed individually by two researchers (RL and AT) with a third arbitrating any disagreements. Reasons for exclusion were recorded. Data were extracted using a standardized form, and the findings were synthesized using thematic analysis.

### 2.4 Outcome measures

We searched for reported processes of risk identification and management as well as differences in approaches for different types of technology. This included:

1. Regulatory guidance on the process of RM in both pre- and post-market stages
2. Reported best practices in risk assessment process
3. Special measures required for innovative technology e.g., AI, CIMD and IoMT
4. Identify gaps in existing frameworks.

**Figure 2:**
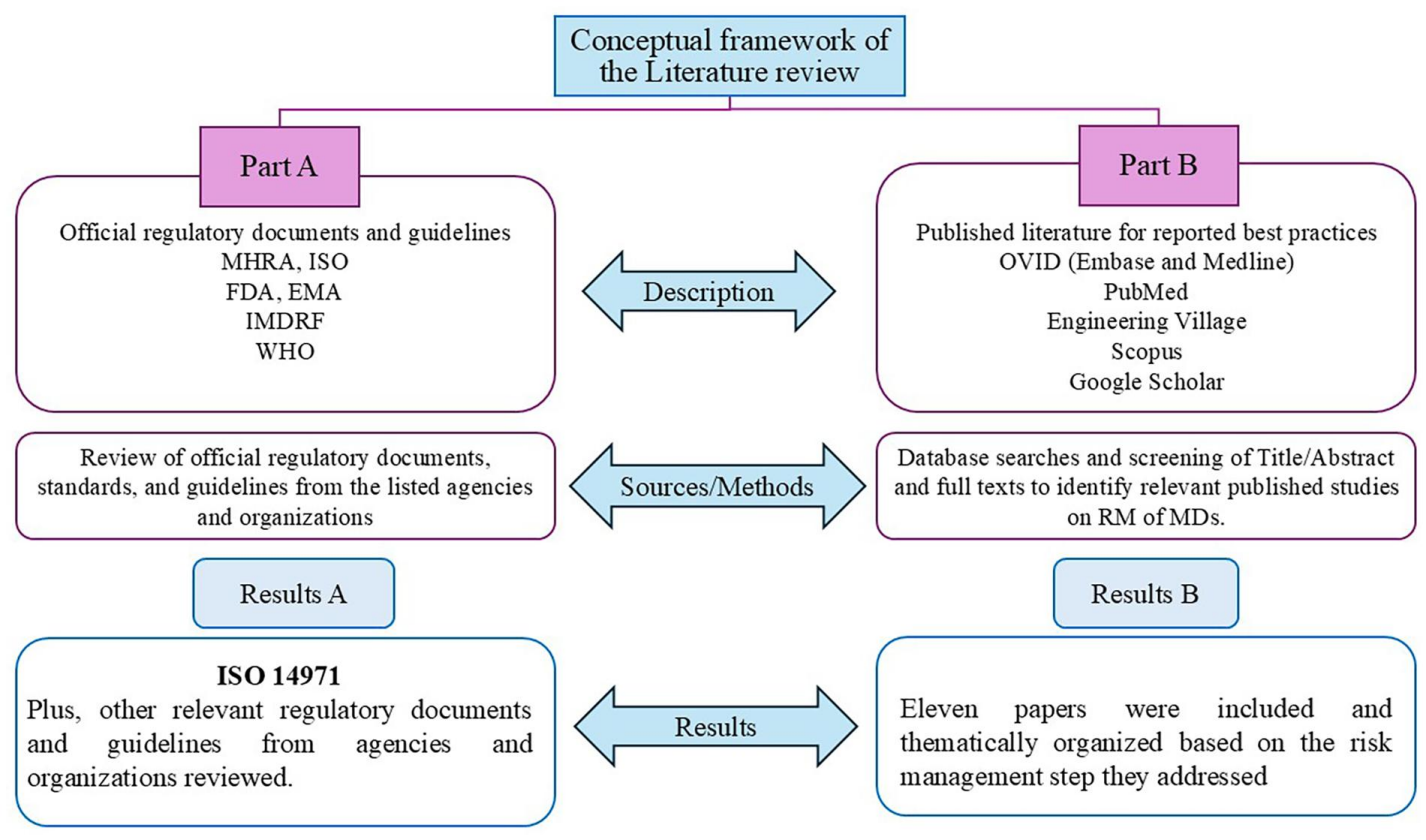
Conceptual framework illustrating the review workflow and evidence synthesis.

A conceptual framework of the review process is presented in Figure 2 to improve clarity and illustrate the relationship between data sources, screening, and synthesis.

## 3 Results

Our initial literature search generated 5943 results. These included published guidelines and research publications. Following abstract review, 147 papers underwent full text review and 12 of these studies fulfilled the eligibility criteria for inclusion in this scoping review. The main reason for exclusion was that the article, document or guideline did not reflect the scope of the study or was aimed at different stakeholders such accreditation organizations, academics or clinical experts.

### 3.1 Step A: published guidelines

Multiple guidelines are published by MHRA, FDA, European Union and IMDRF. In relation to RM and risk assessment, the British Standard EN ISO 14971:2019+A11:2021 is the main document agreed by regulatory bodies (MHRA and FDA) to provide guiding principles on RM of MD (19). Other documents produced by MHRA provide general guidance on specific technology such as IVD or AI and can be used as an adjunct to the ISO 14971 but do not specifically discuss RM process (12, 13). For example, for IVDs, the MHRA advice using end user experience, clear instruction for use, storage and maintenance of the device in reducing risk. Additionally, they provide a number of steps required by the end user in the clinical environment on the maintenance, repair, storage, training required and incident reporting process (12).

Within the European context, Regulation (EU) 2017/745 (MDR) establishes legally binding requirements for risk management, particularly through Annex I, mandating a continuous and systematic process integrated with clinical evaluation and post-market surveillance, without prescribing specific methods (20). In parallel, the International Council for Harmonisation of Technical Requirements for Pharmaceuticals for Human Use Guideline on Quality Risk Management (ICH Q9 R1) provides high-level, cross-sectoral principles and tools to support risk-based decision-making, particularly in the pre-market phase, although it is not specific to medical devices (21). In this context, ISO 14971 provides a key operational framework, translating these expectations into a structured and practical risk management approach for medical devices.

ISO 14971 considers risk as having two components. These are the probability of harm occurring, and the consequences of that harm occurring. The document guides the manufacturer through 4 generalized processes which include (i) identification of hazards, (ii) estimating and evaluating associated risks (iii) finding methods to control those risks and (iv) monitoring the effectiveness of their controls.

Further analysis of these steps and potential shortcomings is presented in Table 2 below. Whilst we consider this guidance document to be invaluable, the application of its principles is challenging and dependable on multiple factors. Table 2 shows this in more detail. Supplementary Table S1 provides a comparative overview of RM standards, frameworks, and guidance for MDs.

**Table 2:** Critical analysis of possible challenging points for MD manufacturers in the process of RM.

Step	ISO 14971:2019+A11:2021	Possible challenges in application of principles
Risk Analysis	The manufacturer must identify the intended use of the MD considering the medical indication, intended population, user profile and environment and operating principles. Reasonably foreseeable misuse must be documented. The characteristics (both qualitative and quantitative) of the MD that can affect safety are identified including their limits. Hazards and hazardous situations must be identified and their relation to safety in both normal and faulty conditions including their consequences. Estimation of risk for each identified hazardous situation. For those situations in which the probability of harm cannot be estimated, its expected consequences should be documented for use in the risk evaluation and control stages.	Identification of the user profile and environment of use can be challenging especially in the home environment. Many assumptions are made in this process creating many challenges and leaving open the possibility of missed processes which cannot be predicted. Limitations still exist on unpredictable use. A hazardous situation can be different for different users. For example, the consequences of an insulin pump malfunctioning on a newly diagnosed diabetic patient with poorly controlled diabetes are different to those of an experienced patient with well controlled blood sugars. This is particularly challenging in the presence of often many unknowns relating to the end user
Risk Evaluation	An estimated risk is allocated to each identified hazardous situation. If the risk is higher than the acceptable risk, then risk control measures should be undertaken.	Acceptable risk is assigned by the manufacturer for their device. In clinical practice, these risks mean something different to different users
Risk Control	Addressing the safety of the design and manufacturing process, providing protective measures within the device, safety information and user training. Verification of implementation of risk control measures and their effectiveness which should be part of the design and development validation within the quality management system. The residual risk is assessed using the criteria for risk acceptability. If the residual risk is not acceptable, then further risk control measures should be taken. If the residual risk cannot be further reduced, a benefit-risk analysis would determine if the benefit of the intended use outweighs the residual risk. Assess possibility of introduction of new risks by the risk control measures themselves. Review of the whole process of risk control	Minimal challenges expected at this step. This is part of quality management system which manufacturers are familiar with. Challenging step with regards to determining what is an acceptable risk and how to mitigate residual risk. This is an essential step which can present challenges depending on the type of technology being assessed and its intended users. This step is expected to be challenging and best assessed at the point of introduction of the new control.
Evaluation of residual risk	Evaluate the overall residual risk posed by the device taking into consideration the criteria above.	This is dependable on the effective steps 1–3
RM review	Prior to commercial distribution of the device ensuring the RM plan has been appropriately implemented and residual risk is acceptable. Appropriate methods for data collection in the post market phase are put in place	Depends on the steps above. These steps are often very challenging due to limitations in staff and funding (depending on the size of the company)
Production and postproduction activities	Information gathering must occur during production and post -production phases. Information must be regularly assessed from the safety and performance aspects looking for previously unrecognised hazards and whether the residual risk is still acceptable. If not, then risk control measures must be again implemented.	Required steps which presents many challenges in real life. This is an essential step with great consequences to the technology and the company producing it.

### 3.2 Step B: published literature

Eleven papers were included in this section. The main step of the RM process addressed was risk analysis, while some papers addressed all the steps. The type of MD was generally not specified, most of the recommendations are applicable to any MD in general according to the authors.

#### 3.2.1 Overall RM

Carden et al. propose a hybrid RM framework combining ISO 14971 and the Project Management Body of Knowledge (PMBOK), aimed at companies that develop MDs (22, 23). Their model integrates PMBOK risk planning, followed by assessment, and monitoring/reporting processes across the lifecycle.

Hopkin et al. highlights that risks increase when users select products unsuited to their circumstances. emphasising the importance of clearly defining target users and ensuring stakeholder collaboration throughout implementation to minimise harm (24).

#### 3.2.2 Risk analysis methods

Freyer et al. identify the FDA Benefit Risk Analysis (BRA) and Multicriteria Decision Analysis (MCDA) as widely used structured approaches for assessing benefit–risk (25, 26). However, since there is no “one size fits all”, they recommend that further research should integrate both qualitative and quantitative methods in BRA, with input from device-specific stakeholders and collaboration with industry in real-world contexts (25).

Tervonen et outline good practices for quantitative BRA (qBRA) using MCDA, particularly for preference-sensitive decisions, providing a roadmap for systematic implementation, and enabling systematic comparison of trade-offs (27).

Kheir et al., through interviews with start-ups, reveal gaps in formal BRA methods and mention risk identification largely managed by senior staff (28). They propose a flexible 4 × 4 RM framework based on ISO 31000 and 14971, emphasizing iterative risk identification and team involvement and incorporating checklists, continuous risk updates, team-wide involvement, and regular register reviews.

Finally, Wu et al. stress the importance of consistent terminology in risk analysis, particularly within complex device environments and global regulatory contexts (29).

#### 3.2.3 Risk evaluation

Ho et al. proposes a framework to incorporate patient preferences into regulatory assessment of MDs (30). The framework outlines how patient preferences can inform regulatory decisions and their value in assessing new technologies and benefit–risk trade-offs.

Expert opinion relevant to AI MDs gathered by Fraser et al. in 2023 states that the required level of clinical evidence should be defined based on the specific application, as well as legal and methodological factors that influence risk such as accountability, transparency, and interpretability (31).

Ho et al. and Fraser et al. inform the risk evaluation stage by supporting value-based judgement and decision-making on risk acceptability, linking the risk level to required clinical evidence and addressing how much evidence is needed to justify acceptance of a given risk.

#### 3.2.4 Risk control

Alhammad et al. propose an evaluation framework for MD–integrated electronic medical record (MDI–EMR) systems that combines MD risk assessment principles with the Human-Organization-Technology fit (HOT-fit) model (32). The framework integrates the HOT-fit model embedding cybersecurity and sociotechnical considerations into control mechanisms.

Bills et al. offer manufacturers an improved approach to meet health and safety RM responsibilities, while proposing the replacement of Failure Mode and effects Analysis with the Risk Traceability Summary (RTS) meeting the requirements of both CFR 820 and ISO 14971 (33).

For Immersive Virtual Reality for At-Home Therapy and Remote Patient Monitoring (HMD-VR) devices Salisbury J., recommends reviewing similar or comparable devices already legally marketed, to facilitate premarket clearance on basis if equivalence as 510(k) pathway (34). The author also highlights the importance of patients understanding the risks associated with HMD-VR and acknowledging cybersecurity, privacy risks and connectivity issues between patient and clinician apps.

#### 3.2.5 Evaluation of residual risk

The literature provides limited explicit guidance on the evaluation of residual risk following implementation of control measures. However, structured benefit–risk frameworks such as BRA and MCDA can support the assessment of whether remaining risks are acceptable in relation to clinical benefits (25–27).

In addition, incorporation of patient preferences may further inform residual risk acceptability, particularly in contexts where trade-offs between risks and benefits are sensitive to stakeholder values (30).

#### 3.2.6 Production and postproduction

Hopkin et al. highlights the importance of monitoring user experience and engagement, particularly where inappropriate use may lead to harm or disengagement from care (24).

Kheir et al. further emphasise the need for continuous risk updates, iterative review processes, and maintenance of risk registers throughout the device lifecycle (28). Fraser et al. underscore the importance of ongoing monitoring for AI-based systems, where evolving risks and changing performance characteristics require continuous reassessment (31).

**Figure 3:**
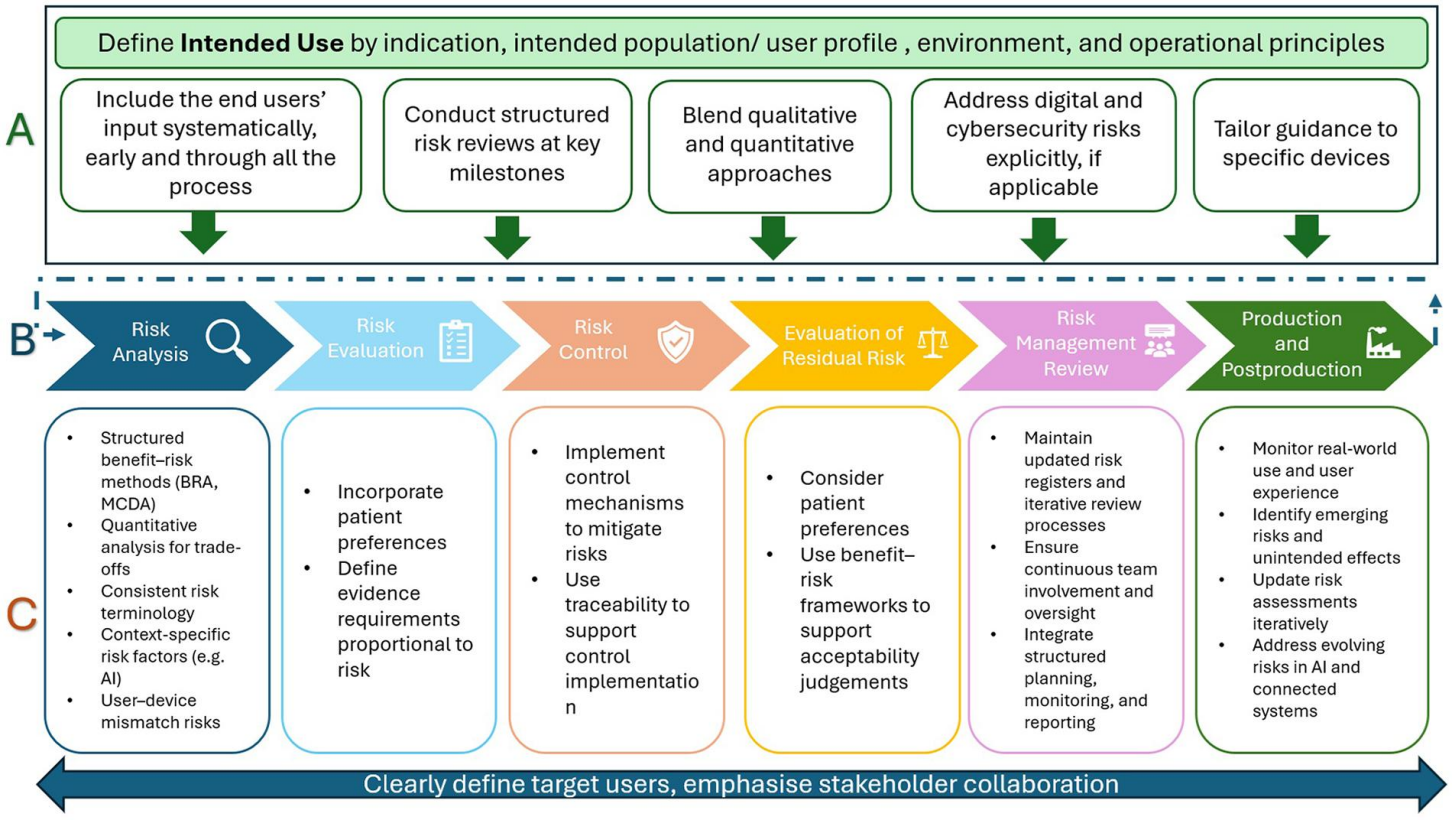
Integration of actionable points and evidence-based best practices in the medical device risk management lifecycle. **(A)** Actionable points proposed by the authors. **(B)** RM process steps as per ISO 14971. **(C)** Literature based best practices mapped across the RM process.

Figure 3 integrates in the RM process the evidence based best practice. (Figures 3B,C).

## 4 Discussion

This study reviewed national and international guidelines and identified key challenges faced by MD manufacturers during the RM process. ISO 14971 provides a structured framework widely recognized by regulators such as MHRA and FDA. The best practices identified in this review fill existing gaps, particularly the predominance of risk analysis methods and the more limited focus on downstream RM stages such as control and post-market monitoring. They extend existing frameworks by providing practical, context-sensitive approaches, especially for emerging technologies, while highlighting areas where further methodological development is still needed. However, significant uncertainties remain regarding end users, use environment, technology characteristics, and acceptable levels of risk.

Predicting misuse of technology and associated potential risks relies on assumptions. Whilst risks and hazards cannot be fully eliminated, some can be mitigated by early use of patient groups, end user involvement and input from clinical teams. In this sense, systematic RM represents not only a mechanism for assuring product safety and compliance but also a cost- and time-saving strategy that can enhance the overall efficiency of MD innovation (16, 17).

### 4.1 Challenges in risk management processes and methods

Most studies focus on risk analysis methods, with fewer addressing control, mitigation, and post-market surveillance. This imbalance may mask the importance of structured RM across all stages. ISO 14971 does not prescribe specific analysis tools, leaving manufacturers to select appropriate methods, while ISO/TR 24971:2020 offers guidance on techniques such as FMEA, FTA, and PHA (35). RTS should not replace conventional RM methods but may serve as a central organizational tool, despite adding steps in the process.

Risk analysis involves identifying hazards and understanding how failures propagate to cause harm t. Hatfcliff J et al. proposed replacing terms such as hazard and harm with “risk concerns” to reflect potential malfunctions affecting device and system performance (10). Though, this may not be widely accepted within the industry, especially in the context of ISO 14971. IEC 80001 (Section A.3) also makes this type of generalisation to include effectiveness and security concerns in addition to patient harm to modify the definition of harm on ISO 14971 (15).

The approaches proposed by Freyer et al. and Tervonen et al. are not mutually exclusive; and given the absence of a “one-size-fits-all” solution underscores the importance of methodological flexibility in RM, guided by device type, decision context, and available evidence (25, 27). Incorporating patient preferences into risk–benefit evaluations, seen in Ho et al.'s framework and Tervonen et al.'s qBRA checklist, aligns with shared decision-making but raises methodological challenges, such as standardizing preference elicitation and ensuring representation (27, 30).

One critical part in RM is the clarity in definition. Already raised by Wu et al, Staworko et al. highlight that ambiguous regulatory definitions can create significant challenges, particularly in the case of IVD tests in the UK (29, 36). Clarity in the regulatory aspect is essential to avoid misinterpretations and to ensure that all stakeholders, i.e., manufacturers, regulators and healthcare providers are aligned in their understanding of risk.

Risk control seeks to reduce the severity and/or probability of harm (15). However, the challenges associated depend heavily on the technology being assessed. For example, the risk associated with a joint implant in an adult differ significantly from those presented by an automated insulin delivery system used by a child with type 1 diabetes. To properly characterise risk, it is essential to fully consider the intended use of the device as well as source of input and effect of output on patient care. This is particularly critical for software and AI-based devices, where the degree of autonomy and clinician involvement varies. The risk is especially crucial in vulnerable patient groups for whom the consequences of incorrect decision-making or device failure could be significant (37).

Several authors proposed adaptations to existing standards (22, 28). Such innovations highlight the need for dynamic, context-responsive RM tools, particularly for smaller firms and digital health innovators.

Finaly, although iterative review is commonly emphasised in post-production activities, it should also be systematically embedded throughout the design and development process. Conducting structured risk reviews at key milestones, such as concept design, feasibility, clinical evaluation, and prior to design freeze, enables timely reassessment of risks and control measures in line with evolving design decisions. This early and continuous approach is particularly important for innovative technologies, where iterative development processes require proactive risk management to avoid downstream impacts on safety, regulatory compliance, timelines, and costs.

### 4.2 Emerging technologies and risks

Emerging technologies, including digital health tools, immersive systems, and integrated platforms, introduce new risks such as user disengagement, interoperability issues, and cybersecurity vulnerabilities (24, 32, 34).

Additionally, AI and Internet of Medical Things based devices, such as CT scanners wearable or implantable monitors (e.g., heart monitors) that collect patient data and provide suggestions for management, introduce distinct challenges related to adaptive algorithms, data-driven decision-making, and evolving risk profiles (38, 39). These interconnected devices offer significant benefits but introduce cybersecurity risks with implications for patient safety and clinical practice (39, 40). AI is identified as a critical technology in the UK Science and Technology Framework. MHRA's white paper outlines a proportionate regulatory approach for AI as a MD (AIaMD), emphasizing transparency, accountability, and safety. The government's pro-innovation AI regulation principles (Feb 2024) acknowledge rapid evolution and incomplete understanding of associated risks, assigning developers responsibility for system safety (13). Existing regulatory frameworks are adapting, but they remain relatively high-level compared to the context-specific guidance required for AI-based systems.

While ISO 14971 provides a structured and widely adopted framework for RM, other frameworks such as those from IMDRF, FDA, and MHRA offer complementary guidance, particularly for software and AI-based devices (12, 13, 37). ISO 14971 is comprehensive but relatively high-level, whereas newer guidance places greater emphasis on context-specific risks such as transparency, adaptability, and cybersecurity. These differences highlight the need for a flexible, combined approach to RM, particularly for emerging technologies.

### 4.3 Integration of human factors and usability in RM

While ISO 9241-112:2025 is not a risk-management standard, its emphasis on clear and effective information presentation can help reduce human-system interaction risks, particularly where misinterpreting system outputs may contribute to harm (41). In this context, ISO 14971 remains the primary structured framework for medical-device risk management, with how to reference providing complementary design guidance rather than procedural direction.

Building on this, EN 62366:2015 introduces a structured usability-engineering process that begins with the definition of user profiles and use specifications, forming the basis for systematic identification of use-related errors and hazardous situations (42). These identified risks are comparatively evaluated against predefined acceptability criteria, followed by the implementation of mitigation strategies embedded within device design and information for safety. The standard further distinguishes between formative evaluation, conducted during development to refine risk controls, and summative evaluation, undertaken to confirm overall usability and residual risk acceptability. Importantly, EN 62366:2015 is product-agnostic, and its structured methodologies are transferable across device types, supporting adaptable and repeatable risk management practices.

As such, EN 62366:2015 supports the development of adaptable and repeatable approaches to managing use-related risks, aligning closely with ISO 14971 while remaining compatible with human-factors design principles such as those described in ISO 9241-112.

The best practices identified address key gaps in the literature. Early stakeholder involvement reduces uncertainty around user behaviour and real-world use, while tailoring guidance to specific devices helps overcome limitations of generic frameworks like ISO 14971 for AI and IoMT. Combining qualitative and quantitative methods supports risk–benefit assessment, and explicitly addressing cybersecurity fills important gaps in traditional RM approaches for interconnected systems.

Drawing on the points raised in our earlier discussion, we developed actionable points, as illustrated in Figure 3A.

These points can be further elaborated, e.g.,:

1. Include the end users’ (patient and stakeholders) input systematically, early and through all the RM process, while ensuring methodological accuracy.
2. Tailoring guidance to specific devices will improve its regulatory relevance and applicability, ensuring that the differences of various technologies are considered in risk assessments.
3. Blending qualitative and quantitative approaches, leveraging the strengths of each to address complex decision contexts.
4. Addressing digital and cybersecurity risks explicitly during the RM process, if applicable.

By implementing these strategies, the MD industry can create more adaptive, device-relevant and patient-centred RM frameworks that align with technological innovation and the safety and effectiveness/efficacy of the innovation for patient health. This ensures that the tools and methodologies used to assess and mitigate risk evolve along with the rapid advancement of innovations and the regulatory landscapes/environments.

This study has some limitations, including the restriction to English-language studies, the application of a 10-year inclusion window, the limited number of included studies, and the potential omission of relevant grey literature. Also, it was limited by the lack of evidence and published literature specifically on best practices.

Many challenges exist with wide variability in technology which alters the process of risk assessment and management. Further work is required to break down the aspects of RM for different device type with clear guidance developed on particular risks e.g., AI, cybersecurity, self-learning modules. Additionally close end user involvement in the process should be sought to achieve systems which would represent real life.

## 5 Conclusions

Many challenges exist in conducting an efficient RM process for MDs. This relates to the variability in technology and its specifications, which is expected to increase with the technology advancements. To address these challenges, several best practices emerge: embedding RM early in the design process, clearly defining intended users and use contexts, and implementing risk control measures as early as possible. In addition, engaging end users and stakeholders across pre- and post-market stages is essential for identifying unknown hazards and improving safety.

Future research should focus on integrating real-world evidence into RM processes, developing clearer links between risk levels and evidence requirements, and establishing practical approaches for meaningful stakeholder involvement in risk management.

## Data Availability

The original contributions presented in the study are included in the article/**Supplementary Material**, further inquiries can be directed to the corresponding author.

## Glossary

MHRA: Medicines and Healthcare products Regulatory Agency. Regulates medicines, medical devices and blood components for transfusion in the UK.
FDA: Federal Drug Administration. Regulates food, drugs, biologics, medical devices, electronics products that give off radiation, cosmetics, veterinary products, and tobacco products.
IMDRF: International Medical Device Regulators Forum. Aims to accelerate international medical device regulatory harmonization and convergence.
BRA: Benefit-risk analysis.
Risk: The likelihood that harm will occur, combined with the potential severity of that harm. *(Adapted from ISO 14971:2019).*
Harm: Any injury to people, or damage to health, property, or the environment. *(Adapted from ISO 14971:2019).*
Hazard: A condition or source that could lead to harm. *(Adapted from ISO 14971:2019).*
Risk Management: A structured approach using policies, procedures, and practices to analyse, evaluate, control, and monitor risks. *(Adapted from ISO 14971:2019).*
Risk Analysis: The systematic process of gathering information to identify hazards and estimate associated risks. *(Adapted from ISO 14971:2019).*
Risk Assessment: The overall process that includes both risk analysis and risk evaluation. *(Adapted from ISO 14971:2019).*
Risk Control: The process of deciding and implementing measures to reduce risks or keep them within acceptable limits. *(Adapted from ISO 14971:2019).*
Risk Estimation: Assigning values to the likelihood of harm occurring and the severity of that harm. *(Adapted from ISO 14971:2019).*
Risk Evaluation: Comparing estimated risks against predefined criteria to determine if they are acceptable. *(Adapted from ISO 14971:2019).*
Medical Device: Any instrument, machine, software, implant, or similar intended for medical purposes in humans, which achieves its primary function without relying on pharmacological, immunological, or metabolic action, though such means may assist its function. *(Adapted from ISO 13485:2016)*.
*In Vitro* Diagnostic (IVD) Device: A device intended for testing specimens from the human body outside the body to provide diagnostic, monitoring, or compatibility information, including reagents, calibrators, control materials, software, and related instruments. *(Adapted from ISO 15189:2022).*
