# Supplementary material for: Reported best practices for effective risk management of medical devices: a scoping review
Source: Front Med Technol. 2026 Jun 24;8:1863808. doi: 10.3389/fmedt.2026.1863808 (PMC13341656; doi:10.3389/fmedt.2026.1863808)
Supplement: Supplementary file 1 [file Supplementaryfile1.docx]

***Supplementary Material***

1. **Supplementary data**

**1.1 Search strategies**

S1.1 OVID MEDLINE

| 1 | Risk Management.mp. or Risk Management/ |
| --- | --- |
| 2 | Risk Assessment.mp. or Risk Assessment/ |
| 3 | Behavioral Risk Factor Surveillance System |
| 4 | Risk Evaluation and Mitigation).mp. [mp=title, book title, abstract, original title, name of substance word, subject heading word, floating sub-heading word, keyword heading word, organism supplementary concept word, protocol supplementary concept word, rare disease supplementary concept word, unique identifier, synonyms, population supplementary concept word, anatomy supplementary concept word] |
| 5 | Risk Reduction Behavior/ |
| 6 | medical device.mp. or "Equipment and Supplies"/ |
| 7 | 1 or 2 or 3 or 4 or 5 |
| 8 | 6 and 7 |
| 9 | limit 8 to english language |
| 10 | limit 9 to yr="2014 - Current" |

1.2 OVID Embase

| 1 | Risk Management.mp. or Risk Management/ |
| --- | --- |
| 2 | Risk Assessment.mp. or Risk Assessment/ |
| 3 | Behavioral Risk Factor Surveillance System |
| 4 | Risk Evaluation and Mitigation).mp. [mp=title, book title, abstract, original title, name of substance word, subject heading word, floating sub-heading word, keyword heading word, organism supplementary concept word, protocol supplementary concept word, rare disease supplementary concept word, unique identifier, synonyms, population supplementary concept word, anatomy supplementary concept word] |
| 5 | Risk Reduction Behavior/ |
| 6 | medical device.mp. or "Equipment and Supplies"/ |
| 7 | 1 or 2 or 3 or 4 or 5 |
| 8 | 6 and 7 |
| 9 | limit 8 to English language |
| 10 | limit 9 to yr="2014 - Current" |

Table 1S provides a comparative overview of RM standards, frameworks, and guidance for MDs.

| **Document / Organization** | **Type / Scope** | **Key Characteristics** | **Advantages** | **Limitations** | **Relationship / Similarities** |
| --- | --- | --- | --- | --- | --- |
| **ISO 14971:2019 (+A11:2021)^(1)^** | International standard | Structured RM lifecycle | Widely accepted; regulator-aligned | High-level; no specific methods | Core RM framework; aligned with MDR |
| **ISO/TR 24971:2020** | Guidance (ISO 14971) | Provides methods (FMEA, FTA, PHA) and examples | Supports implementation | Not mandatory; still generic | Extends ISO 14971 |
| **MHRA Guidance (2021-2023)^(2)^** | Regulatory guidance | Technology-specific recommendations | Context-specific; practical | Not a full RM framework | Complements ISO 14971 |
| **MHRA AI (2024)^(3)^** | Policy guidance | Focus on transparency, accountability, safety | Addresses AI risks | Evolving; limited detail | Aligns with FDA and IMDRF AI guidance |
| **FDA – RM Principles** | Regulatory guidance | Lifecycle RM consistent with ISO 14971 | Regulator-aligned | High-level | Consistent with ISO 14971 and MDR |
| **FDA 2022 CDS** | Device-specific guidance | Focus on CDS risks, transparency, and user understanding | Addresses real-world software risks | Narrow scope | Complements RM frameworks |
| **GMLP (FDA/MHRA/HC 2021)** | AI best practice | AI risk lifecycle | Globally aligned; AI-focused | High-level; not binding | Aligns with IMDRF SaMD |
| **IMDRF SaMD (2024)^(4)^** | International framework | Risk classification and lifecycle for software | Structured approach | Limited implementation detail | Complements ISO 14971 |
| **WHO 2014 (IVD)** | Global guidance | Risk classification for IVDs | Simple; useful for low-resource settings | Limited technical depth | Aligns with regulatory models |
| **EU MDR 2017/745 ^(5)^** | Regulation | Mandates lifecycle RM | Legally enforceable | No methods specified | Relies on ISO 14971 |
| **ICH Q9 (R1) ^(6)^** | International guideline | Risk-based decision principles and tools | Strong conceptual basis | Not MD-specific | Conceptually aligned with ISO 14971 |
| **EN 62366-1:2015^(7)^** | Usability standard | Structured usability process to reduce use errors | Practical; reduces human-factor risks | Limited to usability | Supports ISO 14971 (use-related risks) |
| **ISO 9241-112:2025^(8)^** | Human factors standard | Focus on information presentation and interaction | Improves usability; reduces user errors | Not an RM framework | Complements ISO 14971 and EN 62366 |

References

1. British Standards Institution. BS EN ISO 14971:2019+A11:2021. Medical devices. Application of risk management to medical devices. 2021.

2. Medicines and Healthcare Products Regulatory Agency. Management of in vitro diagnostic medical devices. 2021.

3. Medicines and Healthcare Products Regulatory Agency. Impact of AI on the regulation of medical products. 2024.

4. International Medical Device Regulators Forum. Medical Device Software Considerations for Device and Risk Characterization. 2024.

5. Regulation (EU) 2017/745 of the European Parliament and of the Council, (2017).

6. International Council for Harmonisation of Technical Requirements for Pharmaceuticals for Human Use. Guideline on Quality Risk Management (ICH Q9 R1) 2023.

7. European Committee for Electrotechnical Standardization. EN 62366‑1:2015: Medical devices – Part 1: Application of usability engineering to medical devices. 2015.

8. International Organization for Standardization. ISO 9241‑112:2025: Ergonomics of human‑system interaction — Part 112: Principles for the presentation of information. 2025.
